# A “Regular’s” Opinion of the Pseudo-Homœopath

**Published:** 1882-11

**Authors:** 


### HOMŒOPATHIC PHYSICIAN,

A MONTHLY JOURNAL OF MEDICAL SCIENCE.

"If our school ever gives up the strict inductive method of Hahnemann, we are lost, and deserve only to be mentioned as a caricature, in the history of medicine."—CONSTANTINE HERING.

Vol. II. NOVEMBER, 1882. No. 11.

### A "REGULAR'S" OPINION OF THE PSEUDO-HOMŒOPATH.

Dr. D. W. Cathell, in his little book entitled *The Physician Himself*, speaks thus of the *pseudo-homœopaths:* "When chance brings you into contact with a genuine homœpathist, if you believe him to be a gentleman (*true* homœopathists are usually very respectable and upright), observe all the forms of politeness toward him, and treat him exactly as you would any other gentleman, but ignore him *professionally* and never allow yourself to fraternize with him in the management of a case. But have nothing, emphatically nothing, to do with the pseudo-homœopaths, who masquerade as homœopaths by a display of Hahnemannic nonsense, just as ostrich hunters assume to be ostriches by dressing in that *wise* bird's feathers. Many of these pretenders simulate the genuine by carrying awe-inspiring satchels as guardedly as if an additional shake of the dynamizations they contain might still further increase their potency and cause an explosion. Carefully search the satchel and the pockets of one of these and you will not only find the usual attenuations, triturations, tinctures and globules, and also Lehrman's, Dunham's, Lentz's and Fincke's high dynamizations,* ranging from the 800th away up to the terrific potency of an 86,000th (nonsense! that would not vary the ailments of a fly); but search a little further, and you will also find a full, a very full, supply of Warner's, Schieffelin's,

* Dr. Cathell is in error here. These fellows seldom or never carry these excellent preparations, for two reasons: (1), they do not know enough to use them; and (2), because the allopaths do not approve of them.

Sharpe & Dohme's, and other varieties of sugar-coated granules of Morphia, Quinia, Arsenicum, Belladonna, Elaterium, Colocynth, etc.

"Be not startled if you also find a hypodermic syringe and a bottle of Magendie's solution—damning witness of his lack of moral sense and lack of honesty, and of his want of faith in what he professes. Respect every sincere believer in a false system, no matter how great his error, but let the finger of scorn point forever at each and every double-dealing hypocrite who, as an advertisement of himself, villifies and sneers at '*old-school*' remedies, while *slyly using Opium* to relieve pain, Chloral to induce sleep, Quinia to arrest fever, and all our other prominent agents *just as we do,* in full doses, and crediting the good they do to Homœopathy.

"There is also another self-adjusting variety, much less numerous, thank Heaven! than the last, who, chameleon-like, are all things to all men, who actually *offer* to practice any exclusive system people wish. These are not as bad as the last, for they are at least honest in their announcement.

"But what would you think of a clergyman whose love of gold and lack of scruple would allow him to vary his principles *at will,* and preach *anything* you wished, whether strictly Catholic lecture or an ultra-Protestant discourse, an orthodox Hebrew sermon, a fiery Mohammedan philippic, or an out-and-out infidel harangue? He might believe in one or more, but he could not believe in all. Show a decent respect for the conscientious homœopath, but shun, as you would the plagues of Egypt, the *bogus* and the *anything you please* fellows, who use the name simply as a cloak because it pays to use it."

As our liberal friends attach so much weight and importance to all "regular" utterances, we hope they will ponder over this philippic; it is severe, but true, in its denunciation of quacks.

Judging from the above quotation, and many similar opinions, it would seem that the famous resolution of the Institute seeking allopathic recognition will be as fruitless as it is degrading.

